# Zinc bioaccessibility of foodstuffs after *in vitro* study in children with illnesses

**DOI:** 10.29219/fnr.v69.11032

**Published:** 2025-12-26

**Authors:** Úrsula García-Conde, Miguel Navarro-Moreno, Beatriz Navajas-Porras, Daniel Hinojosa-Nogueira, Adriana Delgado-Osorio, Sergio Pérez-Burillo, Miguel Navarro-Alarcón, Silvia Pastorizal, Konstantinos Douros, José-Ángel Rufián-Henares

**Affiliations:** 1 Department of Nutrition and Bromatology, Faculty of Pharmacy, University of Granada, Granada, Spain; 2 Institute of Nutrition and Food Technology (INyTA), Institute for Biosanitary Research (IBS), University of Granada, Granada, Spain; 3 Pediatric Allergy and Respiratory Unit, 3rd Department of Pediatrics, School of Medicine, ‘Attikon’ University Hospital National and Kapodistrian University of Athens, Athens, Greece

**Keywords:** Zn availability in the large bowel, children with metabolic disorders, cooking techniques under which foods are usually consumed, health disorders in childhood

## Abstract

**Background:**

Childhood is a life stage particularly sensitive to malnutrition, including obesity, celiac disease, and food allergies. Zn is an essential element in development, metabolism, and the regulation of inflammatory processes and oxidative stress.

**Objective:**

Zn bioaccessibility (Zn-BA), an indicator of the fraction of Zn available for intestinal absorption, is commonly evaluated through *in vitro* digestion models. This study applied a novel *in vitro* digestion–fermentation method to assess Zn-BA in various raw and cooked foods using fecal inocula from children with gluten-related disorders (GRD-CH), obesity (OB-CH), and allergy/intolerance to cow’s milk protein (AICM-CH).

**Results:**

The results showed that Zn bioaccessibility in the large intestine (Zn-BALI) values were significantly lower in these clinical groups compared to healthy children (*P* < 0.001). Mean Zn-BALI values in the large intestine of all foods in GRD-CH are significantly higher (34.7 ± 28.8%) than those determined in OB-CH (29.6 ± 30.1%) and AICM-CH (26.7 ± 30.4%) (*P* < 0.001). For allergic children, Zn-BALI in animal foods was significantly lower than in plant foods and in both plant and animal foods in the other children’s groups (*P* < 0.05). Zn-BALI in animal foods cooked in liquid media (frying/boiling) was significantly higher than when cooked with hot air (roasting/grilling).

**Conclusion:**

In children, the studied diseases diminished the Zn-BALI, which could negatively affect their appropriate long-term development. In sick children, the higher Zn-BALI in celiac children is probably related to differences in gut microbiota composition, as well as to different metabolites and ligands obtained by the fermentation processes, a fact that should be addressed in future studies.

## Popular scientific summary

- In this study, we applied a novel *in vitro* digestion/fermentation method to multiple raw and cooked foods by metabolization with fecal inoculate from sick children (celiac, obese, and allergic children) to evaluate the bioaccessibility of zinc (Zn-BA) in the large intestine.
- Zn-BA in the large intestine in children varies with disease, probably because of the differences in gut microbiota composition and different metabolites and ligands obtained by the fermentation processes, a fact that should be addressed in future studies.

Childhood represents a life stage particularly sensitive to nutritional imbalances, whether because of excessive energy intake leading to overweight and obesity or to inadequate nutrient intake associated with disorders such as gluten-related diseases and allergy or intolerance to cow’s milk proteins. Zn is an essential trace element required for normal growth and development. It acts as a cofactor in numerous enzymatic systems involved in macronutrient metabolism, immune and inflammatory regulation, and oxidative stress control (1, 2). For Zn to exert these physiological roles, it must first be released from the food matrix and subsequently absorbed in the intestine. Therefore, the *in vitro* digestion/fermentation model employed in this study serves as an effective preliminary tool for assessing Zn bioavailability, providing an essential screening step before *in vivo* studies in experimental animals or humans (3).

Celiac disease is an enteropathy resulting in damage to the intestinal microvilli and alterations in the resident microbiota, decreasing the intestinal barrier function (4) and micronutrient absorption, with occurrences of Zn deficiencies (5). It is a genetic autoimmune disease with lifelong treatment using gluten-free diets, resulting in deficiencies in fiber and Zn, which may be associated with the modification of the gut microbiota and production of ligands that influence Zn bioaccessibility (Zn-BA) (6).

In obesity, it has been shown that serum Zn levels were lower in obese women than in lean controls (7). Besides, decreased body Zn status and serum levels have been reported in obese children and adults (8). In addition, it has been described that Zn decreases neuroinflammation and improves cognitive function in obesity (9).

On the other side, immunoglobulin-E-mediated cow’s milk allergy is the most prevalent allergy in infancy, affecting between 2 and 3% of infants and young children (10). In the persistence of cow’s milk casein allergy, the consumption of hydrolyzed formulas based on rice, whey, or soy is recommended, because of its positive modulation of the immune system and intestinal barrier function through the regulation of the gut microbiota (11). On one hand, food allergies are conditioned by the diet in early childhood, especially in children with familial predisposition to atopic manifestations (12), so nursing mothers should delay the consumption of highly allergenic foods such as dairy by-products.

The mechanisms underlying the relationship between the metabolic disorders examined in this study and Zn-BA are not yet fully understood. Nevertheless, Zn is an essential trace element whose metabolism is subject to strict homeostatic regulation, which may be disrupted under pathological conditions such as celiac disease, obesity, and cow’s milk protein allergy. Consistent with this, previous studies have reported reduced Zn-BA in individuals with celiac disease and obesity (5, 6). This reduction likely results from alterations in gut microbiota composition and metabolic activity, impairing Zn release during colonic fermentation. Furthermore, these disorders are often accompanied by intestinal inflammation and mucosal dysfunction, which can further limit Zn solubilization and potential absorption.

Zn bioavailability is also adversely affected by several anti-nutritional factors, including phytates, polyphenols (such as tannins), dietary fibers, oxalates, and excessive concentrations of Ca, Fe, or Cu (13–16). Whether these inhibitory compounds exert differential effects in children with the metabolic disorders studied remains unknown.

Our research group demonstrated that Zn-BA in the large intestine of the foods considered in the present work in healthy children (HE-CH) was higher than in healthy adults (12), because of the Zn needs for optimal growth in childhood. This study is a continuation of the previous one, evaluating Zn-BA in the large intestine among unhealthy children, using as a control group the HE-CH of the previous study.

Zinc absorption depends on its solubility at the absorption site, body status, growth demands, and pathological state (16–20). In the intestine, only soluble chelates would increase the Zn solubility and absorption (18). Moreover, Zn absorption from the diet and foodstuffs (and therefore corresponding bioavailability/bioaccessibility) is related to chemical forms, food type, and transformations during processing and digestion (16, 21, 22, 23).

Camara et al. (24) reported that *in vitro* assessments of elemental dialysis and solubility show a good correlation with mineral bioaccessibility. On the other hand, bioavailability refers to the fraction of a nutrient, such as Zn, that is absorbed and converted into a metabolically active form in the body, typically evaluated in *in vivo* studies in experimental animals or, preferably, in humans under conditions that accurately reproduce human physiology. However, human bioavailability studies are complex, requiring measurement of dietary Zn absorption, accounting for the fraction excreted in feces and urine, and, in some cases, using invasive methods to assess transformation into an active form or accumulation in specific organs. In contrast, bioaccessibility, as assessed in the present study, estimates the fraction of Zn released from the food matrix, and solubilized and available for absorption in short periods of time (24–48 h) (25). This study includes 55 different foods corresponding to 159 food samples, considering the culinary preparation under which they are typically consumed. For this reason, in this work, we employed *in vitro* bioaccessibility studies as an initial screening, allowing the evaluation of the fraction of Zn solubilized in the intestinal lumen after *in vitro* digestion and fermentation, which would be potentially available for absorption. After this *in vitro* method, a continuous bioavailability assessment system as the Simulator of the Human Intestinal Microbial Ecosystem (SHIME) (26) could be used.

Mineral bioaccessibility during digestion is critical for promoting absorption and overall element bioavailability (27). We hypothesize that Zn-BA is influenced by both domestic cooking methods and the gut microbiota composition of children with gluten-related disorders (GRD-CH), obesity (OB-CH), or allergy/intolerance to cow’s milk proteins (AICM-CH). This study aimed to determine Zn-BA in foods prepared using common home-cooking techniques after *in vitro* digestion and fermentation with fecal inocula from GRD-CH, OB-CH, and AICM-CH, and to compare these values with those obtained from healthy controls (16). We hypothesized that the amount of Zn released and solubilized would differ among groups because of disease-specific interactions between gut microbiota and the food matrix, providing insights for targeted nutritional interventions and preventive strategies. Previous studies have demonstrated that gut microbiota can modulate mineral absorption (28, 29), that cooking methods influence Zn-BA (16, 30, 31), and that nutrient absorption is altered in GRD-CH, OB-CH, or food allergies (32–34).

## Materials and methods

### General reagents and equipment

Cysteine, sodium dihydrogen phosphate, sodium sulfide, resazurin, salivary *α*-amylase, pepsin from porcine, and bile acids (porcine bile extract) were from Sigma-Aldrich (Darmstadt, Germany). Pancreatin from porcine pancreas was purchased from Alpha Aesar (Lancaster, UK). Milli-Q water, reagent grade (Millipore, Bedford, MA, USA), was used. A single-element 1,000 mg/L standard solution of Zn from Merck was used for the preparation of working aqueous standard solutions by appropriate dilution with 2–1% HNO_3_–HCl (Merck).

A microwave digester (Multiwave 5000 with Rotor 24HVT50, Anton Parr GmbH, Graz, Austria) was used for sample mineralization. Inductively coupled plasma mass spectrometry (ICP-MS/MS; Agilent 8900 Triple Quadrupole ICP-MS, Agilent Technologies Inc., Santa Clara, CA, USA) was used for Zn measurements.

### Food samples and culinary techniques

A total of 159 samples of commonly consumed foods were analyzed. The list of foodstuffs and the culinary techniques used for cooking can be checked as supplementary material (16, 35) (Supplementary Table S1). For their culinary processing, six cooking techniques were used: raw form, roasting, frying, toasting (only for whole-grain bread and bread), boiling, and grilling. Foods were analyzed in the form that they are normally consumed by the population. For food cooking, the food/medium ratios and times were adapted (3). For frying, heating at 180˚C (8 min) with an extra virgin olive oil-to-food ratio of 5:1 was used. For grilling, heating between 220 and 250˚C (3 min) with an extra virgin olive oil-to-food ratio of 0.5:1 was utilized. For food broiling, heating at 180˚C (10 min) was used. In this study, the zinc content of the cooking oils (especially extra virgin olive oil used in frying and grilling) was not considered. For boiling, heating at 100˚C (20 min) with a water-to-food proportion of 5:1 was used. For roasting, a fourth level (900 W, 3 min) of the toaster (Grunkel TS140H) was used. The ratio of samples and their treatments, as well as the utensils used for preparation, can be found elsewhere (3). Next, before the samples were used for analysis, they were homogenized and finally stored at −80˚C (in a nitrogen atmosphere).

### Stool samples from GRD-CH, OB-CH, and AICM-CH groups

Different groups of GRD-CH (*n* = 10), OB-CH (*n* = 10), and AICM-CH (*n* = 10) were considered and compared with HE-CH (16). All children were 8–10 years old. A common exclusion criterion was taking antibiotics or probiotics in the previous 3 months. Lean and obese children diagnosed with chronic gastrointestinal disorders or being on a special diet were excluded from the study. The BMI of the celiac, lean, and milk-allergic children was comprised between the 5th and 85th percentile for their gender, height, and age. For the obese group, BMI was above the 95th percentile for sex, weight, and age.

Stool sample containers were provided to children at a hospital in Athens (Greece) by the pediatric department. The protocol followed for the collection of feces and the requirements to be fulfilled by the participants are described elsewhere (16). The study was conducted according to the guidelines of the Declaration of Helsinki and approved by the Ethics Committee of the University of Granada (Protocol Code 1080/CEIH/2020). It was also approved by the Scientific Committee of the University Hospital of Ioannina (Protocol Number 382, Date 4 June 2020, Decision number 10/3-6-2020), the Scientific Committee of the University Hospital ‘Attikon’ (Decision Number: 546/1-10-2020), and the Scientific Committee of the University Hospital of Patras (Decision Number: 360/22-7-20). Informed consent was obtained from the legal representatives of the children involved in the study.

Fecal samples were collected in sterile bottles (0.6 L volume). Next, avoiding direct contact with the fecal sample, two anaerobic gas generator sachets (AnaeroGen Compact, Thermo Scientific) were located inside the reported bottles. A home refrigerator was used for storing samples at the hospitals, and transportation was performed in a cooler bag within 24 h. Next, at the laboratories, the bottles were opened under anaerobic conditions (anaerobic chamber with 80% N_2_, 10% CO_2_, and 10% H_2_) and the stools were then mixed with a 20% vol/vol water/glycerol solution. The final storage was done at −80°C. For the feces’ processing, samples were shipped to the Spanish laboratory for no longer than 2 months under previously established conditions (in dry ice at −80°C) (3).

### In vitro digestion and fermentation method

To mimic physiological processes in the human gut, all food samples were subjected to an *in vitro* digestion process previously described elsewhere (26, 35) with three phases (buccal, stomach, and gut phase). Then, simulated salivary fluid (with salts and *α*-amylase (75 U/mL) was put into the falcon tube with food (1:1, w/v), leaving the obtained mixture in oscillation (37°C, 2 min) (Supplementary Table S2). Immediately, mimicking the gastric juices (in salts and pepsin (2,000 U/mL), 10 mL of simulated gastric fluid was added (Supplementary Table S2). The mixture was left in oscillation under determined conditions (37°C, 2 h, pH = 3). Finally, 20 mL of the simulated intestinal fluid (67.2 mg/mL pancreatin) was added (Supplementary Table S2). The mixture was left in oscillation under standardized conditions (37°C, 2 h, pH = 7) (3). Next, once the bowel phase was ended, tubes were kept in ice (to stop enzymatic reactions) and centrifuged (3,500 rpm for 10 min). Then, two fractions were obtained: the digestion supernatant, with soluble Zn available for hypothetical absorption in the small bowel (Zn bioaccessibility in the small intestine [Zn-BASI]), which was kept in 1 mL tubes at −80°C until analysis; and the solid pellet.

Then, an *in vitro* fermentation was performed using fecal samples from participants in the study (GRD-CH, OB-CH, and AICM-CH). Therefore, the solid residue (0.5 g) plus 10% of the digestion supernatant of foods (both obtained in the digestion phase) was fermented under anaerobic conditions (anaerobic workstation with 80% N_2_, 10% CO_2_, and 10% H_2_) under oscillation at previously reported conditions (37°C, 20 h) (3, 26). The composition of the fermentation medium was cysteine (312 mg/L), peptone (14 g/L), resazurin (0.1%, v/v), and hydrogen sulfide (312 mg/L), from which 7.5 mL was added to the fermentation tube. Inoculum was made from stools previously described. Each fecal sample was mixed with phosphate buffer saline (pH = 7.0, 33% concentration). Next, 2 mL of inoculum was added to the fermentation tube (each food sample was fermented three times, once for each digestion), and to get anaerobic conditions, N_2_ was bubbled, leaving a transparent solution (contrary to the pink color reached by the existence of O_2_). Then to avoid microbial reactions, tubes were stored on ice and immediately centrifuged (3,500 rpm for 10 min). Again, two fractions were obtained: the fermentation supernatant, with soluble Zn available for hypothetical absorption in the large bowel (Zn bioaccessibility in the large intestine [Zn-BALI]); and the solid pellet, as non-fermented fraction excreted with feces. Both fractions (supernatant and solid pellet obtained in the fermentation stage) were kept under identical conditions as reported earlier until analysis (3).

After the *in vitro* method, three different fractions were obtained: 1) digestion supernatant as the fraction available for hypothetical Zn absorption in the small bowel, expressed as Zn-BASI; 2) fermentation supernatant as the accessible fraction of Zn available in the large bowel (in the proximal colon), expressed as Zn-BALI; and 3) fermentation solid residue as the Zn fraction not available for absorption and excreted with feces, expressed as non-bioaccessible Zn fraction. The sum of the Zn levels of these three fractions allowed the determination of the Zn amount present in the analyzed foods (3).

For all foodstuffs studied, the *in vitro* method was done in triplicate. Zn concentrations corresponding to the different fractions were expressed in ppm (mg Zn/kg, fresh weight).

### Mineralization of digestion and fermentation supernatants and fermentation solid pellets of foodstuffs and analysis of Zn

For the foodstuffs studied, a previously described procedure was used (16). Samples were weighed into borosilicate tubes, followed by the addition of 3 mL of 65% HNO_3_ and disposal into microwave Teflon digestion vessels, which were then placed in the rotor of a microwave digester. Mineralization was performed under previously optimized conditions (16). The mineralized samples were appropriately diluted with Milli-Q water (40 mL) to obtain the analytical dissolution. In this solution, Zn was measured by ICP-MS/MS using an Internal Standard Kit (Ge, Ir, Rh Sc; ISC Science, batch 20210712).

In each of the batches, four blanks were prepared. The measurements were carried out using the linear calibration method and in triplicate for each of the samples analyzed.

Prior to the determination of Zn concentrations using the ICP-MS technique, we carried out a study of the analytical parameters of the procedure used. To check the accuracy and precision of the method, bovine muscle powder nº 8414 and citrus leaves powder nº 1515 were used as reference standards certified in Zn (National Institute for Standards and Technology [NIST], Gaithersburg, MD, USA). The values obtained for the limit of detection (0.42 µg/L), accuracy (144 ± 2.5 and 12.5 ± 0.68 μg/g for certified levels of 142.0 ± 14.0 and 12.7 ± 0.85 μg/g of reported reference materials, respectively), and precision (relative standard deviation lower than 10%) of the method demonstrate the suitability of the method for the measurement of Zn. The calculated recoveries for Zn ranged between 98.5 and 101.3% (16).

### Statistical analysis

The statistical analysis of the data obtained was carried out with Statistical Package for the Social Sciences (SPSS version 28.0, IBM, IL, USA). Data were expressed as mean Zn values ± standard deviation. For figures, Zn values were expressed as mean ± Standard Error of the Mean (SEM) in order to facilitate the observation of the experimental groups of samples between which significant differences were established. The existence of statistically significant differences was set to a *P*-value lower than 0.05 (*P* < 0.05). For the Zn-BA values obtained, firstly, the existence of homogeneity of variances for the different experimental groups of samples described (e.g. different groups of children) was checked by means of Levene’s test (*P* > 0.05), and secondly, the normal distribution of the data was checked by means of the Kolmogorov-Smirnov test (*P* > 0.05). When the homogeneity of variances and the normal distribution of the data were fulfilled, the parametric method of Student’s *t*-test was used in the analysis of variance (ANOVA). However, if the homogeneity of variances or the normal distribution of the data was not met (*P* < 0.05), the Kruskal-Wallis nonparametric test was used, and the Mann-Whitney test was used as the nonparametric method in the comparison between two groups of specific samples (e.g. between Zn-BA values for the cooking techniques).

## Results and discussion

### Zn-BALI of foods in GRD-CH

**Fig. 1:**
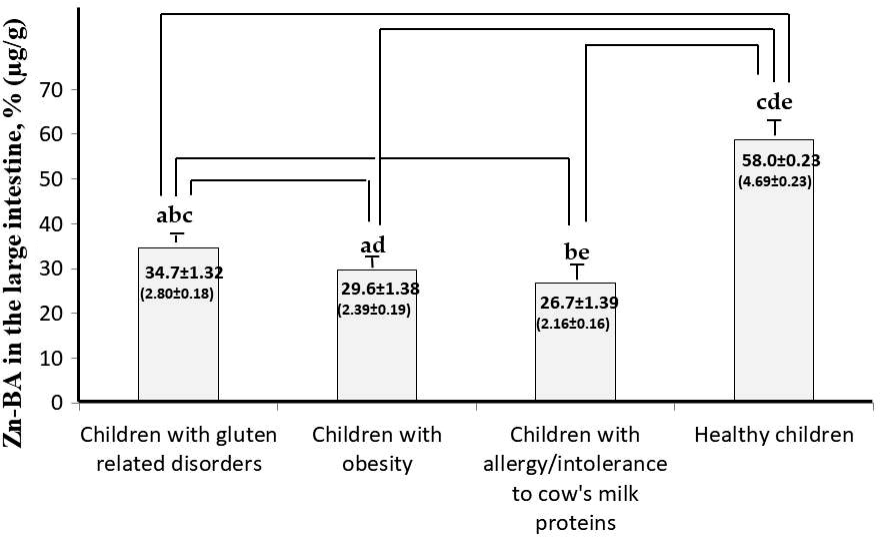
Mean Zn bioaccessibility values (%, microg/g) in the large intestine of unhealthy children compared with healthy children (HE-CH) (16). Bars with identical lowercase letters indicate statistically significant differences between child groups (*P* < 0.05).

Figure 1 shows mean Zn-BALI values of all foods in GRD-CH, which are significantly higher than those determined in OB-CH and AICM-CH, and lower than those in HE-CH (*P* < 0.001) (16). The decrease in Zn-BA of all children with illnesses in comparison to controls may be related with the atrophy of the intestinal microvilli, worsening nutritional absorption and leading to Zn deficiencies (5, 36, 37).

**Fig. 2:**
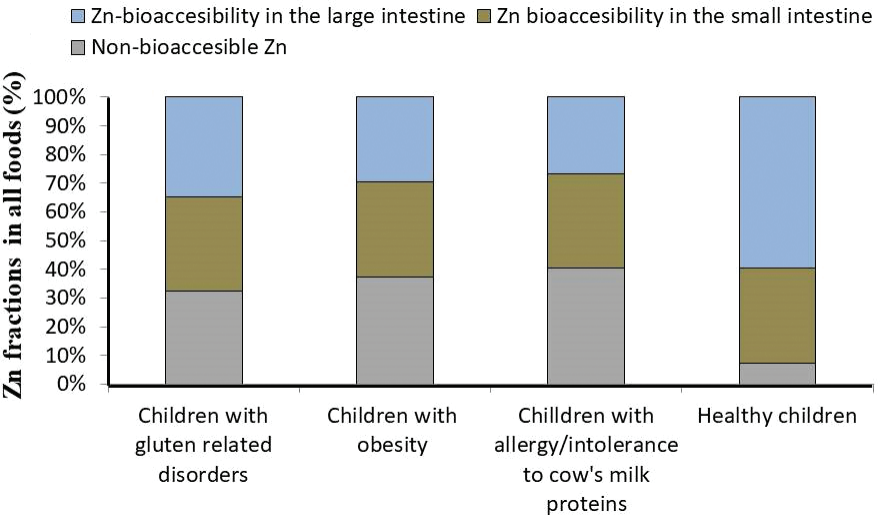
Non-bioaccessible and bioaccessible Zn fractions in foods in the small (16) and large intestine of unhealthy and healthy (16) children.

Figure 2 shows the distribution of Zn between bioaccessible and non-bioaccessible fractions across different children’s groups, expressed as a percentage. Zn-BA values in the small intestine were previously published elsewhere (16).

More than 50% of untreated celiac patients are Zn-deficient because of the described atrophy, chelation of Zn with fatty acids, elevated fecal loss because of protein-losing intestinal pathology, or high Zn use in enterocyte turnover (38). Some of the symptoms of celiac disease, such as the characteristic decreased growth and anorexia, are related to Zn deficiency. One possible explanation for this decrease could be that following gluten-free diets in GRD-CH leads to a decrease in the consumption of fermentable fiber, with a consequent decrease in butyric acid-producing microorganisms (39); this would lead to a decrease in the formation of soluble chelates that are bioaccessible with Zn. In celiac patients, there are variations in the gut microbiota and lumen compared to healthy subjects, and the microbial proteolytic pathways involved in gluten detoxification in the inflamed duodenum are impaired (40).

Some researchers reported that strict gluten-free diets in celiac, after 1 year of maintenance, counteract Zn deficiencies and do not require long-term supplementation (28, 41). In the Canadian population, it was found that subjects on a gluten-free diet did not have deficient Zn intakes, because of the consumption of mineral and vitamin supplements in the last 30 days prior to the study (41). Despite this, it would be advisable to recommend that GRD-CH treated with gluten-free diets consume foods with higher Zn content and Zn-BA, such as nuts, dairy products, vegetables, lamb, legumes, and beef (Table 1). Furthermore, in gluten-free diets, rice is usually the most consumed grain, for which a low Zn intake has been indicated (19, 42).

**Table 1:** Mean Zn contents (µg/g, fresh weight) and Zn bioaccessibility values in the large intestine (Zn-BALI; %; ± standard deviation) of plant- and animal-based foods of unhealthy and healthy children*

Food group	Zn (mg/g, ppm)	Zn-BALI in GRD-CH (%)	Zn-BALI in OB-CH (%)	Zn-BALI in AICM-CH (%)	Zn-BALI in HE-CH^#^ (%)
Plant-based foods					
Nuts^†^	12.8 ± 4.80	52.7 ± 31.4^a^	39.7 ± 28.2^ab^	18.8 ± 29.9^c^	66.6 ± 25.3^bc^
Cereals^†^	6.47 ± 2.00	33.5 ± 28.6^a^	33.8 ± 28.8^b^	34.3 ± 36.6^c^	56.4 ± 27.3^abc^
Fruits^†^	7.63 ± 4.79	36.7 ± 28.7^ab^	27.8 ± 32.1^ac^	27.3 ± 29.5^d^	48.2 ± 31.2^bcd^
Vegetables^†^	8.75 ± 3.08	36.9 ± 26.0^ab^	29.5 ± 29.3^acd^	34.8 ± 29.3^ce^	62.5 ± 20.8^bde^
Legumes^†^	6.83 ± 2.51	41.6 ± 35.3^a^	22.4 ± 29.2^b^	18.9 ± 30.6^c^	57.8 ± 26.7^abc^
Oils^†^	1.75 ± 0.70	0.46 ± 0.58	0.21 ± 0.28	20.2 ± 33.9	1.29 ± 1.60
Beverages^†^	5.99 ± 8.67	0.35 ± 0.17	0.23 ± 0.26	1.32 ± 5.58	0.64 ± 0.63
Others^†^	6.60 ± 1.29	38.2 ± 30.4	24.2 ± 30.1	16.6 ± 26.5	39.9 ± 39.5
Animal-based foods					
Dairy products^†^	11.0 ± 5.76	37.1 ± 22.1^a^	30.8 ± 33.2	25.6 ± 34.2^ab^	46.9 ± 37.8^b^
Chicken^†^	5.04 ± 1.13	48.6 ± 38.8^a^	31.5 ± 38.4^b^	15.7 ± 30.0^c^	62.0 ± 28.8^abc^
Beef^†^	13.7 ± 2.05	30.7 ± 23.4^a^	42.5 ± 24.3^b^	16.2 ± 20.6^bc^	45.8 ± 24.6^ac^
Salmon^†^	6.73 ± 2.16	31.8 ± 25.0^ab^	42.7 ± 26.7^cd^	9.36 ± 20.3^ace^	55.7 ± 25.6^bde^
Cod^†^	5.62 ± 1.93	37.6 ± 34.1^a^	43.5 ± 30.7	16.2 ± 24.6^b^	58.6 ± 32.5^ab^
Egg^†^	8.97 ± 2.36	14.5 ± 12.6^ab^	20.6 ± 19.4^c^	36.1 ± 27.1^acd^	40.4 ± 27.6^bd^
Lamb^†^	6.97 ± 2.80	45.2 ± 25.8	41.5 ± 32.9	12.4 ± 29.3	31.2 ± 32.4
Pork^†^	7.40 ± 0.48	37.4 ± 31.7	49.2 ± 27.4	9.20 ± 23.7	42.7 ± 32.4

* Children with gluten-related disorders (GRD-CH); children with obesity (OB-CH); children with allergy/intolerance to cow’s milk proteins (AICM-CH); healthy children (HE-CH).

† The existence of rows labeled with the same superscript lowercase letters for Zn-BALI values in every food category for different children groups denotes the existence of statistically significant differences (*P* < 0.05).

# Data from healthy children have been previously published (16).

Zn-BALI is higher in celiac children than in the remaining unhealthy children. This finding could be related to the different composition of the gut microbiota in each disease, and to the different metabolites/ligands obtained through fermentation processes that may enable the formation of more bioaccessible Zn chelates. Abdukhakimova et al. (43) reported that *Bifidobacterium longum* deserves special attention in future studies as a biomarker of celiac disease. Others (44) found no differences in the composition of the gut mucosal microbiota in celiac compared to healthy controls. Soheilian-Khorzoghi et al. (45) reported a significant decrease in the abundance of *Bifidobacterium* spp., *Lactobacillus* spp., and *Firmicutes* in celiac patients, probably because of increased consumption of beef and beans. Other researchers (46) have reported similar findings in celiac subjects, along with increased abundances of the genera *Prevotella* and *Clostridium*, and *Escherichia coli*. The reduction in *Lactobacillus* and *Bifidobacterium* populations, and consequently the lower production of short-chain fatty acids (SCFAs) derived from carbohydrate and protein fermentation (28, 29), may be linked to the decrease in Zn-BALI observed in GRD-CH. This relationship likely reflects a reduced formation of soluble SCFA–Zn complexes, which can facilitate Zn solubilization and absorption. However, this potential mechanism requires confirmation through future studies.

Within plant foods, in cereals, fruits, vegetables, and legumes, the Zn-BALI values of GRD-CH were significantly lower than those of HE-CH (*P* < 0.001; Table 1). Additionally, for nuts, fruits, and vegetables, the Zn-BALI values in GRD-CH were significantly higher than those determined in OB-CH (*P* < 0.05). In foods of animal origin (Table 1), such as chicken, beef, salmon, cod, and whole egg, Zn-BALI levels were significantly lower in GRD-CH than those measured in HE-CH (*P* < 0.05). In dairy products, salmon, and whole eggs, Zn-BA values in GRD-CH were significantly higher than those measured in AICM-CH (*P* < 0.05).

**Fig. 3:**
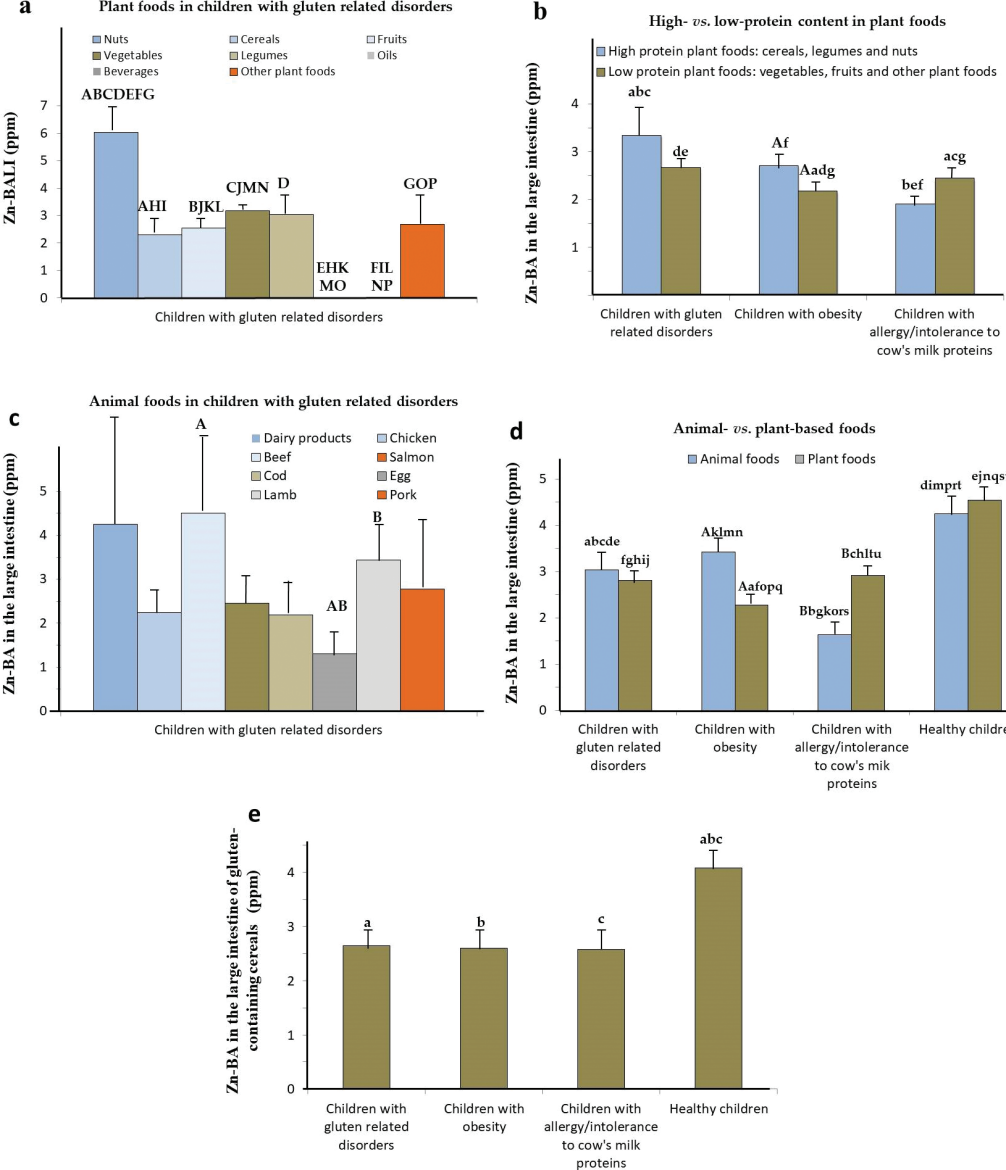
Mean Zn bioaccessibility values in the large intestine (Zn-BALI; µg/g fresh weight) of children. (a) Zn-BALI for children with gluten-related disorders (GRD-CH) in plant-based foods. (b) Zn-BALI for unhealthy children in plant-based foods according to their protein content. (c) Zn-BALI for GRD-CH in animal-based foods. (d) Zn-BALI for unhealthy and healthy children (HE-CH) (16) in animal- and plant-based foods. (e) Zn-BALI for unhealthy and HE-CH (16) in gluten-containing cereals. Bars with identical uppercase or lowercase letters indicate statistically significant differences (*P* < 0.05).

The plant-based foods in GRD-CH showed the highest Zn-BALI compared to the values found for OB-CH and AICM-CH (Table 1). In addition, Zn-BALI was found at high levels in nuts, vegetables, and legumes (Fig. 3a).

In celiac children, low-protein plant-based foods showed significantly higher Zn-BALI values than those with a lower and higher protein content (*P* < 0.05; Fig. 3b) found for obese and allergic children, respectively (*P* < 0.05; Fig. 3b). In addition, the Zn-BALI of plant-based foods with a high protein content in the GRD-CH group is significantly higher than that of the low-protein foods in OB-CH and AICM-CH (*P* < 0.05). This could be explained because vegetal proteins and their amino acids would facilitate the formation of soluble chelates with Zn in GRD-CH, as was previously reported (18). Additionally, previous studies have shown that diets richer in plant-based proteins promote greater SCFA production and are associated with higher abundances of *Bifidobacterium* spp. (28), which may partly explain the higher Zn-BALI observed for plant-derived foods with higher protein content in GRD-CH. In animal-based foods, the highest Zn-BALI in GRD-CH corresponded to beef, dairy products, and lamb, and the lowest to whole egg and salmon (Fig. 3c).

In Fig. 3d, we observe that the values of Zn-BALI in GRD-CH (for plant-derived foods) are significantly higher and lower than those in OB-CH and HE-CH, respectively (*P* < 0.001; Fig. 3d). Additionally, in animal foods, they were significantly higher and lower than those determined in AICM-CH and HE-CH, respectively (*P* < 0.05; Fig. 3d). It is possible that, in celiac patients, the intestinal dysbiosis characteristic of the disease (46, 47) underlies the differences observed in Zn-BA both among the groups of children with metabolic disorders and between these groups and healthy controls. In this regard, Naseri et al. (47) reported an increased *Firmicutes*-to-*Bacteroidetes* ratio in celiac individuals.

For GRD-CH, the highest Zn-BALI values in cereals (Supplementary Table S3) were measured in wholemeal breakfast cereals and biscuits, and the lowest in rice and whole rice.

As shown in Supplementary Table S4, Zn-BALI for most vegetables of unhealthy children is significantly lower than that corresponding to HE-CH, and specifically for orange in fruits (Supplementary Table S4).

The Zn-BALI values in GRD-CH are significantly higher in gluten-containing cereals (38.2 ± 28.1%) compared to gluten-free ones (5.02 ± 4.52%) since it only included rice/brown rice, as was also found in obese and HE-CH. Despite rice being the most consumed grain in gluten-free diets (36), we found that the Zn-BALI is markedly low, showing a risk of nutritional Zn deficiency in celiac children.

In celiac children, Zn-BALI in gluten-containing cereals is only significantly lower than that determined in HE-CH (*P* < 0.05; Fig. 3e). In line with this, others (38) reported that more than 50% of untreated celiac patients are Zn deficient.

### Zn-BALI of foods in obese children

Zn-BALI values for all foods in OB-CH are significantly lower than those determined in celiac and HE-CH (*P* < 0.001) (16). This decrease in Zn-BALI (Fig. 1) may be one mechanism leading to the diminishment in body Zn status described in obese/overweight children and adults (7, 8).

Others suggested that there is a negative correlation between body mass index and serum Zn levels (8), and that excess body fat decreases the blood concentration of Zn and zinc-*α*2-glycoprotein (1). In addition, Zn has been shown in experimental animals to promote gut microbiota biodiversity (48, 49), improving intestinal barrier integrity and decreasing intestinal inflammation and translocation (50). Squizani et al. (9) indicate that Zn supplementation in Wistar rats partially reduced the harmful effects of a cafeteria diet but did not protect obese animals from intestinal dysbiosis. However, others (48) reported that consumption of Zn biofortified wheat-based diets by rooster specifically increased microbial *β*-diversity with enhancement of SCFA-producing lactic acid bacteria and positive restructuring of gut microbiota.

The significant decrease in Zn-BALI in obese compared to HE-CH raises the need for further studies of supplementation of their diets with bioavailable forms of Zn to improve their body status. It has been suggested that Zn modulates the gut microbiota (49), and in the Zn-deficient situations posed by obesity, its bioaccessibility is reduced, as we have found in the present study for the Zn-BALI. Future studies should be therefore planned to determine the components of the gut microbiota, and which microbes (through the fermentative processes of specific food components) enable the development of metabolites that act as Zn ligands, forming insoluble chelates that decrease the observed Zn-BALI.

It has been suggested that intestinal dysbiosis may contribute to several processes associated with obesity, including altered nutrient absorption, as observed in this study for Zn-BA. In obese children, dysbiosis typically manifests as reduced microbial diversity and an altered *Firmicutes*-to-*Bacteroidetes* ratio, characterized by an overrepresentation of *Firmicutes*, conditions that are closely linked to inflammation and increased energy harvest (51). The lower Zn-BALI observed in OB-CH may therefore be related to enhanced Zn uptake by pathogenic microorganisms within the dysbiotic intestinal environment, a hypothesis that warrants further investigation.

In plant-based foods (Table 1), the Zn-BALI of nuts, cereals, fruits, vegetables, and legumes in the obese group is significantly lower than in healthy controls (*P* < 0.001). Additionally, Zn-BALI values in OB-CH in vegetables are significantly lower than those determined in AICM-CH (*P* < 0.05). In animal foods, only in chicken and salmon, Zn-BALI values are significantly lower in obese than in HE-CH (*P* < 0.05). Additionally, Zn-BALI in beef and salmon for the obese children is significantly higher than in allergic children (*P* < 0.05).

**Fig. 4:**
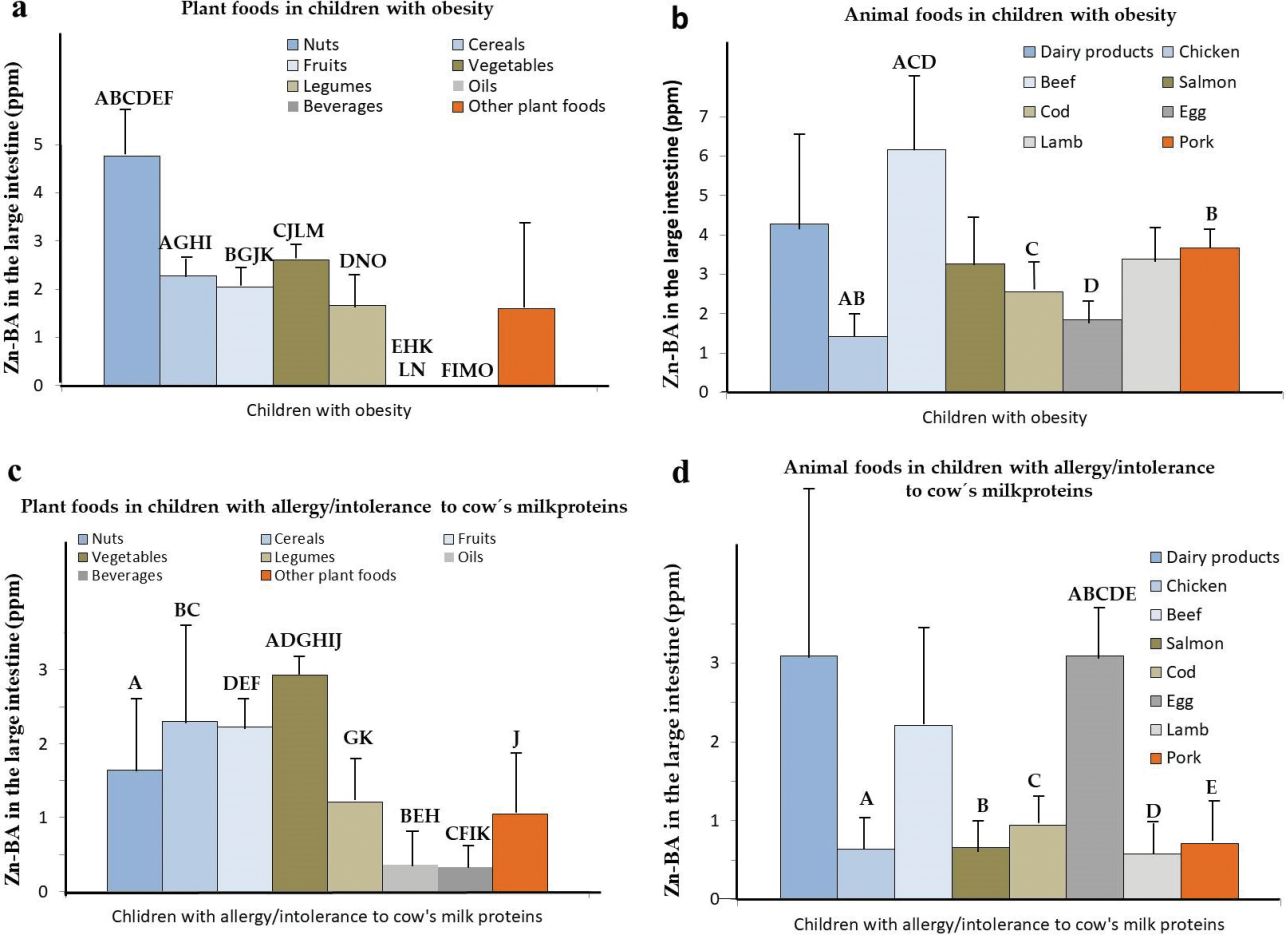
Mean Zn bioaccessibility values in the large intestine (Zn-BALI; µg/g fresh weight) of unhealthy children. (a) Zn-BALI for children with obesity in plant-based foods. (b) Zn-BALI for children with obesity in animal-based foods. (c) Zn-BALI for children with allergy/intolerance to cow’s milk (AICM-CH) proteins in plant-based foods. (d) Zn-BALI for AICM-CH in animal-based foods. Bars with identical uppercase letters indicate statistically significant differences (*P* < 0.05).

In plant-based foods, the highest values of Zn-BALI in OB-CH correspond to nuts, vegetables, and cereals (Fig. 4a; *P* < 0.001). Specifically, Zn-BALI in nuts is significantly higher (*P* < 0.05) than in almost all remaining plant food groups.

In low-protein plant-based foods, Zn-BALI values of OB-CH are significantly lower than those found for higher protein plant-based foods (*P* < 0.05; Fig. 3b), as well as those observed in both high- and low-protein foods for celiac children (*P* < 0.05; Fig. 3b). It is noticeable that in OB-CH, especially for low-protein plant foods, Zn-BALI is diminished to a greater extent compared to the other two diseases. This again could be related to the specific composition of the gut microbiota of OB-CH (51). Additionally, the high protein content, together with carbohydrates – particularly those present in cereals and legumes – provides the necessary substrates for the Maillard reaction, promoting the formation of compounds such as furosine, methylfurfural, and hydroxymethylfurfural (52). These compounds, often classified as fiber-associated components, can facilitate the generation of metabolites such as SCFAs, which are known to form soluble Zn–SCFA chelates, thereby enhancing Zn-BA in the colon. Moreover, Maillard reaction products have been reported to modulate the composition of the intestinal microbiota, potentially influencing Zn solubilization and absorption (53).

In OB-CH, the highest Zn-BALI values in cereals (Supplementary Table S3) are found in breakfast cereals and biscuits, while the lowest are in rice/whole rice. In vegetables, the lowest Zn-BALI values are measured in potatoes (Supplementary Table S4).

In plant-based foods, the highest Zn-BALI levels are found for nuts in obese children (Fig. 4a). In animal-based foods, the highest values of Zn-BALI correspond to beef and dairy products and the lowest to chicken and whole eggs (Fig. 4b).

In plant-based foods, Zn-BALI values are significantly lower than those determined in animal-derived foods (*P* < 0.05; Fig. 3d) for obese children, contrary to findings for allergic children (16). Likewise, Zn-BALI for both animal- and plant-based foods in obese children is significantly lower than those for HE-CH (*P* < 0.001; Fig. 3d). Additionally, Zn-BALI in the plant-based foods of OB-CH is significantly lower than in GRD-CH (*P* < 0.05). In foods of animal origin, Zn-BALI in OB-CH is significantly higher than in AICM-CH (*P* < 0.05; Fig. 3d). This differential behavior of Zn-BALI in plant-based foods for OB-CH may be linked to specific alterations in their intestinal microbiota composition, an aspect that warrants further investigation. Moreover, obese children typically consume diets characterized by higher energy density, lower polysaccharide and dietary fiber content, and elevated levels of animal protein, saturated fats, and simple sugars. These dietary patterns are consistently associated with a reduction in *Bacteroidetes*, an increase in the *Firmicutes*-to-*Bacteroidetes* ratio, and a higher relative abundance of *Proteobacteria* and SCFA-producing bacteria (28).

### Zn-BALI of foods in children with allergy/intolerance to cow’s milk proteins

Figure 1 shows Zn-BALI values in the large intestine of foods in allergic children, which are significantly lower than in celiac and HE-CH (*P* < 0.001) (16). In young adults allergic to cow’s milk mediated by IgE, others found a decrease in final height and in nutritional intake of Zn (54). Seppa et al. (55) reported in infants with cow’s milk allergy the negative effects of cow’s milk consumption on growth, which recovered when replaced by soy- or hydrolyzed whey-based formulas. Other authors (56) did not find any significant differences in growth between children with cow’s milk protein allergy who were fed therapeutic formulas and those who were breastfed. Additionally, in children with food protein-induced gastrointestinal allergies, those who did not consume hypoallergenic formulas did not meet the nutritional Zn requirements, despite mineral supplementation (57). Moreover, in children aged 0–2 years consuming a complementary cow’s milk exclusion diet, a deficiency in Zn (58) was described. Contrarily, other researchers (59) reported in infants that consumption of both a cow’s milk exclusion and an unrestricted cow’s milk diet facilitated an intake exceeding the nutritional Zn requirement.

IgE-mediated and non-IgE-mediated cow’s milk protein allergy is associated with intestinal dysbiosis (56, 60, 61). In recent years, the need to design healthier gut microbiota-based diets for the treatment of cow’s milk protein allergy has been considered (10), which should consider which gut microbiota profiles are associated with increased Zn bioavailability.

In children with cow’s milk protein allergy, a reduction in *Bifidobacterium* abundance and an increase in *Clostridium* and *Escherichia–Shigella* have been reported (56). As mentioned earlier, the decrease in fermentative microorganisms such as *Bifidobacterium* could lead to lower production of SCFAs and, consequently, to a reduction in Zn-BALI, as observed in AICM-CH in the present study – a finding that warrants further investigation in future research.

In plant-based foods, the levels of Zn-BALI in nuts, cereals, fruits, vegetables, and legumes for allergic children are significantly lower than in HE-CH (*P* < 0.001; Table 1). In animal-based foods, Zn-BALI values are significantly lower in chicken, beef, salmon, cod, and whole egg samples in allergic children than in the HE-CH group (*P* < 0.05). Furthermore, Zn-BALI values found for AICM-CH in dairy products and beef samples are significantly lower than in GRD-CH and OB-CH, respectively (*P* < 0.01).

In plant-based foods (Fig. 4c), the highest Zn-BALI values in AICM-CH correspond to vegetables, cereals, and fruits. Additionally, Zn-BALI of vegetables is also significantly higher than in nuts, fruits, legumes, oils, beverages, and other plant foods (*P* < 0.05; Fig. 4c; Table 1).

In AICM-CH, the highest Zn-BA values in cereals (Supplementary Table S3) are measured in breakfast cereals and wholegrain breakfast cereals, and the lowest in rice. In vegetables, Zn-BALI in potatoes for AICM-CH is significantly higher (*P* < 0.01) than in GRD-CH and OB-CH. This should be deeply studied in future research by its possible correlation with the specific gut microbiota of allergic children.

In animal-based foods, there are also statistically significant differences in Zn-BALI for the allergic group between whole egg, with higher values, and chicken, salmon, cod, lamb, and pork (Fig. 4d; *P* < 0.001). The highest values are found for dairy products and whole egg.

Zn-BALI in plant-based foods in AICM-CH is significantly higher than in animal-based ones (*P* < 0.05; Fig. 3d). Likewise, the values of Zn-BALI in both animal and plant foods for allergic children are significantly lower than in HE-CH (*P* < 0.001, Fig. 3d). The Zn-BALI of animal foods in AICM-CH is significantly lower than in plant- and animal-based foods of the remaining unhealthy and HE-CH (*P* < 0.05). This result could be related to the gut dysbiosis induced by allergy to cow’s milk proteins (60) and the associated inflammatory processes, which could lead to allergies to other animal food proteins, and therefore to a more pronounced decrease in Zn-BALI in animal-based foods.

### Zn-BALI of foods depending on home culinary techniques in unhealthy children

Table 2 presents the Zn-BA values for plant- and animal-based foods according to the home cooking techniques used (frying, raw form, roasting, toasting, boiling, and grilling).

**Table 2:** Mean Zn (µg/g, fresh weight) and Zn bioaccessibility values in the large intestine (Zn-BALI; %; ± standard deviation) of plant- and animal-based foods depending on the home culinary techniques used of unhealthy and healthy children*

Home culinary technique	Zn (µg/g, ppm)	Zn-BALI in GRD-CH (%)	Zn-BALI in OB-CH (%)	Zn-BALI in AICM-CH (%)	Zn-BALI in HE-CH^#^ (%)
Plant-based foods					
Frying^†^	9.10 ± 3.75	25.7 ± 26.8^a^	23.9 ± 28.6^b^	36.8 ± 29.9^c^	59.1 ± 23.6^abc^
Raw form^†^	6.88 ± 4.27	36.1 ± 29.7^a^	28.3 ± 30.8^ab^	25.9 ± 31.7^c^	53.3 ± 31.9^bc^
Roasting^†^	10.1 ± 5.32	36.2 ± 29.4^a^	28.8 ± 31.6^b^	26.7 ± 29.6^c^	64.1 ± 20.^4abc^
Toasting^†^	7.00 ± 1.21	55.2 ± 21.6^a^	47.1 ± 25.7^b^	41.6 ± 35.9	74.0 ± 5.22^ab^
Boiling^†^	6.77 ± 2.88	33.5 ± 27.8^a^	25.5 ± 28.3^b^	32.6 ± 32.8^c^	62.0 ± 20.3^abc^
Grilling^†^	7.42 ± 3.53	38.4 ± 27.7^a^	27.5 ± 28.6^b^	24.9 ± 27.6^c^	60.1 ± 19.3^abc^
Animal-based foods					
Frying^†^	9.75 ± 2.05	48.5 ± 27.6^ab^	49.5 ± 32.9^cd^	17.4 ± 23.2^ace^	65.8 ± 23.6^bde^
form^†^	9.39 ± 7.34	40.7 ± 33.1	37.2 ± 33.1	28.7 ± 36.6^a^	52.2 ± 32.2^a^
Roasting^†^	7.52 ± 3.10	18.4 ± 16.2^a^	25.6 ± 17.1^b^	16.0 ± 24.9^abc^	46.0 ± 17.4^c^
Boiling^†^	8.20 ± 4.31	54.8 ± 32.8^a^	57.4 ± 29.6^b^	18.0 ± 30.0^abc^	71.0 ± 21.0^c^
Grilling^†^	7.67 ± 3.78	17.5 ± 17.1^ab^	17.2 ± 17.9^c^	15.2 ± 28.3^ad^	46.0 ± 17.9^bcd^

* Children with gluten-related disorders (GRD-CH); children with obesity (OB-CH); children with allergy/intolerance to cow’s milk proteins (AICM-CH); healthy children (HE-CH).

† The existence of rows labeled with the same superscript lowercase letters for Zn-BALI values in different home culinary techniques for animal- and plant-based foods for different children groups denotes the existence of statistically significant differences (*P* < 0.05).

# Data from HE-CH have been previously published (16).

**Fig. 5:**
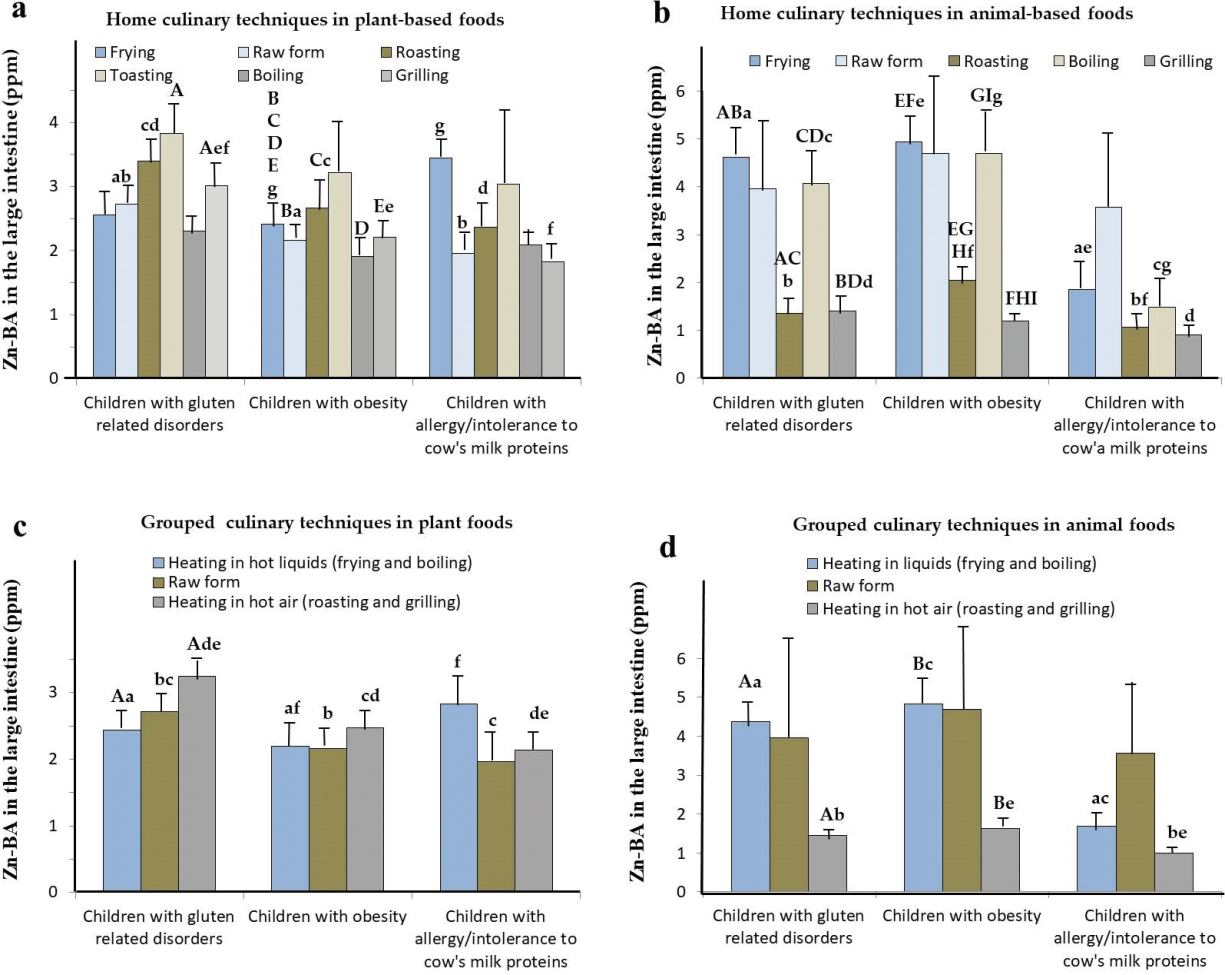
Mean Zn bioaccessibility values in the large intestine (Zn-BALI; µg/g fresh weight) of unhealthy children according to home culinary technique. (a) Zn-BALI in plant-based foods by cooking method. (b) Zn-BALI in animal-based foods by cooking method. (c) Zn-BALI in raw plant foods compared with those heated by hot liquids or hot air. (d) Zn-BALI in raw animal foods compared with those heated by hot liquids or hot air. Bars with identical uppercase or lowercase letters indicate statistically significant differences (*P* < 0.05).

In *celiac children*, the Zn-BALI measured for all culinary techniques used in plant-based foods (Table 2) was significantly lower than in HE-CH (*P* < 0.05), except for the raw form. Furthermore, Zn-BALI in the raw form, roasting, and grilling was significantly higher than in OB-CH and AICM-CH (*P* < 0.05; Table 2; Fig. 5a: for Zn-BALI). In animal foods (Table 2), only frying and grilling showed significantly lower Zn-BALI values than in HE-CH (*P* < 0.05). In contrast, Zn-BALI for frying, roasting, boiling, and grilling was significantly higher than in AICM-CH (*P* < 0.05; Table 2).

In *obese children*, the measured Zn-BALI of plant-based foods was significantly lower for all culinary techniques used with respect to HE-CH (*P* < 0.05; Table 2). However, in animal-based foods (Table 2), only in frying and grilling techniques, Zn-BALI was significantly lower than in HE-CH (*P* < 0.05). Conversely, Zn-BALI (Table 2, Fig. 5b) for frying, roasting, and boiling was significantly higher than in AICM-CH (*P* < 0.05).

In *allergic children*, for plant-and animal-based foods, Zn-BALI was significantly lower for almost all culinary techniques compared to HE-CH (*P* < 0.05; Table 2).

From all foods (plant-based foods: Fig. 5a; and animal-based foods: Fig. 5b), there are significant differences (*P* < 0.05) in Zn-BALI in both vegetal- and animal-based foods according to the cooking technique used. In plant-based foods, Zn-BALI values in raw, roasted, or grilled foods were significantly higher in children with celiac disease than in those with obesity or cow’s milk protein allergy (Fig. 5a; *P* < 0.05). In celiac and obese children, Zn-BALI values for frying and boiling were significantly higher than in foods submitted for roasting and grilling (Fig. 5b). In children with cow’s milk protein allergy, Zn-BALI values (µg/g) were significantly lower in foods prepared by frying, roasting, boiling, or grilling compared with those observed in children with celiac disease (Fig. 5b). Overall, for celiac and obese children, Zn-BALI in fried and boiled foods is higher than in roasted and grilled foods. These results are probably related to the specific gut microbiota composition of the different groups of unhealthy children considered. Thus, although there are practically no differences in Zn-BALI levels on foods of plant origin for allergic children (neither compared to other children’s groups nor culinary techniques), in the case of foods of animal origin, higher Zn-BALI values were obtained, especially for fried, roasted, or boiled foods.

We also studied Zn-BALI levels of unhealthy children by grouping cooking techniques according to whether foods were heated in hot liquid media (frying/boiling) or by hot air (grilling/roasting) and compared them with the raw form. Plant-based foods (Fig. 5c) showed significantly higher (*P* < 0.05) Zn-BALI values in celiac children compared with obese children across all three cooking techniques, and compared with allergic children for the raw, roasted, and grilled forms. In animal-based foods (Fig. 5d), for unhealthy children, Zn-BALI in foods heated in liquid media was significantly higher than those heated by hot air (Fig. 5d). In celiac and obese children, Zn-BALI of foods heated in liquid media and hot air was significantly higher than in allergic children (*P* < 0.05; Fig. 5d).

Consequently, the less aggressive culinary techniques for animal-based foods in liquid media (frying/boiling) give rise to higher Zn-BALI in all unhealthy and healthy (16) children, which should be possibly related to their gut microbiota (48, 53, 62). It is possible that intense roasting and grilling destroy/modify the ligands that form soluble complexes with Zn, exerting a negative effect on Zn-BALI in the large intestine. These last effects should be confirmed in future studies to identify the ligands and the direct action that intense heating exerts on them and on their ability to form chelates with Zn, as well as their relationships with the composition and functionality of the gut microbiota (26, 53, 63).

Consequently, it is important to understand Zn-BA to identify home cooking methods that enhance its intestinal absorption (64) and help mitigate Zn deficiencies, which are especially common among children in developing countries.

An important limitation of the work is the small number of samples for some foods and/or home cooking technologies. Another limitation is the unavailability of information on the dietary and growth patterns of the studied children, as well as biochemical laboratory parameters. However, the large number of food samples (*n* = 159) is a strength that allows evaluating the differences in Zn-BALI between children groups. On the other hand, the reported results were obtained without accounting for the mass balance, which represents a methodological limitation that should be addressed in future research. Another important limitation is that the specific composition of the colonic microbiota in the groups of children with the metabolic disorders studied has not yet been characterized, nor has the potential influence of their habitual dietary intake on microbial composition been evaluated – crucial aspects that warrant investigation in future studies.

## Conclusion

In conclusion, we have found that the Zn-BA of all studied foods in the large intestine of celiac (34.7 ± 28.8%), obese (29.6 ± 30.1%), and allergic children (26.7 ± 30.4%) was lower than that observed in HE-CH (16). Such a decrease could adversely affect long-term development and exacerbate the progression of these health disorders. Zn-BA in the large intestine of celiac children was higher than that of obese and allergic children, probably because of differences in gut microbiota composition and in the metabolites or ligands generated during fermentation – an aspect that should be further investigated in future studies. In obese children, the lower Zn-BA observed (compared to healthy controls) could represent one of the mechanisms contributing to the reduced body Zn status reported by other authors. In allergic children, the lower Zn-BALI values observed in animal-based foods compared with plant-based foods may promote intestinal dysbiosis and inflammatory processes, potentially predisposing them to additional food protein allergies.

Overall, previous research indicates that children affected by these metabolic disorders exhibit an increased *Firmicutes*-to-*Bacteroidetes* ratio and a decreased abundance of *Bifidobacterium* and other lactic acid-producing microorganisms. This microbial imbalance is commonly associated with enhanced intestinal inflammation and reduced SCFA production, both of which may diminish Zn solubilization through decreased formation of Zn–SCFA complexes.

Future studies should therefore assess the relationship between dietary intake, the specific composition of the colonic microbiota, and SCFA production in these pediatric populations, as well as their direct correlation with Zn-BA in both raw and cooked foods. In addition to their anti-inflammatory properties, SCFAs are linked to specific microbial phyla in the colon, whose contribution to Zn solubilization should be elucidated.

Finally, an additional relevant finding of this study is that cooking animal-based foods in liquid media (e.g. frying or boiling) appears to be less detrimental than dry-heat methods (e.g. roasting or grilling), resulting in higher Zn-BALI values in children with metabolic disorders.

## Supplementary Material


