# Supplementary material for: Zinc bioaccessibility of foodstuffs after in vitro study in children with illnesses
Source: Food Nutr Res. 2025 Dec 26;69:10.29219/fnr.v69.11032. doi: 10.29219/fnr.v69.11032 (PMC12767679; doi:10.29219/fnr.v69.11032)
Supplement: Supplementary file 1 [file FNR-69-11032-s1.docx]

**Zinc bioaccessibility of foodstuffs after *in vitro* study in children with illnesses**

***Supplementary material***

*Table legends*

*Table S1.* Food samples considered in the study, either in their raw form or after being submitted to different culinary processes (**n**: number of samples) (16).

*Table S2.* Concentration (mM/L) and composition of salts in the stock solutions, and their respective concentrations (mM/L) in the simulated oral, gastric, and intestinal fluids used in the *in vitro* model (3).

*Table S3*. Mean Zn contents (µg/g, fresh weight) and bioaccessibility values in the large intestine (Zn-BALI; %;±standard deviation) of cereal by-products of unhealthy and healthy children^*^.

*Table S4*. Mean Zn contents (µg/g, fresh weight) and bioaccessibility values in the large intestine (Zn-BALI; %;±standard deviation) of vegetables and fruits of unhealthy and healthy children^*^.

*Table S1.*

| Food type | Food group | Food  item | Culinary processes or raw form | *n* |
| --- | --- | --- | --- | --- |
| Animal | Meat | Chicken, beef, lamb, pork | Frying, roasting, boiling, grilling | 16 |
|  | Fish | Salmon | Frying, roasting, boiling, grilling, raw form | 5 |
|  |  | Cod | Frying, roasting, boiling, grilling | 4 |
|  | Dairy | Butter | Frying, raw form | 2 |
|  |  | Milk (cow milk, fermented cow milk) | Raw form | 2 |
|  |  | Gouda cheese | Frying, roasting, grilling, raw form | 4 |
|  | Egg | Egg | Frying, roasting, boiling, grilling | 4 |
| Vegetal | Fruits | Apple, banana, orange, grapes, plum, peach | Frying, roasting, grilling, raw form | 24 |
|  |  | Olive | Raw form | 1 |
|  | Cereals | Regular bread, whole-grain bread | Frying, toasting, raw form | 6 |
|  |  | Penne, whole-grain penne | Boiling | 2 |
|  |  | Rice, whole-grain rice | Boiling | 2 |
|  |  | Biscuits, whole-grain biscuits | Raw form | 2 |
|  |  | Breakfast cereals, whole-grain breakfast cereals | Raw form | 2 |
|  | Nuts | Nuts mixture, peanuts | Frying, roasting, raw form | 6 |
|  | Legumes | Lentils, kidney beans | Roasting, boiling, grilling | 6 |
|  | Vegetables | Zucchini, pepper, carrot, eggplant, onion, cauliflower, tomato, spinach, garlic, cabbage | Frying, roasting, boiling, grilling, raw form | 50 |
|  |  | Lettuce | Raw form | 1 |
|  |  | Tubers (potato, sweet potato) | Frying, boiling, grilling | 8 |
|  | Oils | Sunflower oil, olive oil | Frying | 4 |
|  | Beverages and infusions | Beer, red wine, coke (regular coke, light coke), coffee (regular coffee, instant coffee) | Crude form | 6 |
|  | Others | Dark chocolat, hazelnut spread | Raw form | 2 |

*Table S2.*

| Salt composition | Salt concentration in stock solution | Salt concentration in simulated oral fluid (pH 7) | Salt concentration in simulated gastric fluid (pH 3) | Salt concentration in simulated intestinal fluid (pH 7) |
| --- | --- | --- | --- | --- |
| KCl | 0.5 | 15.09 | 6.9 | 6.8 |
| KH_2_PO_4_ | 0.5 | 1.35 | 0.9 | 0.8 |
| NH_4_CO_3_ | 1 | 13.68 | 25 | 85 |
| NaCl | 2 | - | 47.2 | 38,4 |
| MgCl_2_.(H_2_O)_6_ | 0.15 | 0.15 | 0.12 | 0.33 |
| NH_4_(CO_3_)_2_ | 0.5 | 0.06 | 0.5 | - |
| CaCl_2_.(H_2_O)_2_ | 0.3 | 1.5 | 0.15 | 0.6 |
| HCl | 6 | 1.1 | 15.6 | 8.4 |

*Table S3.*

| Cereal by-product | Zn (μg/g, ppm) | Zn-BALI in  GRD-CH (%) | Zn-BALI in  OB-CH (%) | Zn-BALI in  AICM-CH (%) | Zn-BALI in  HE-CH^#^ (%) |
| --- | --- | --- | --- | --- | --- |
| Bread^†^ | 7.33±3.54 | 33.6±27.3 | 30.4±23.5^a^ | 25.8±30.8^b^ | 61.2±21.5^ab^ |
| Bread whole meal^†^ | 9.08±3.31 | 31.9±18.9^a^ | 29.7±24.1^b^ | 34.1±32.1 | 61.4±20.4^ab^ |
| Penne^†^ | 2.89±0.41 | 8.40±12.6 | 20.5±35.5 | 26.4±45.4 | 45.2±12.2 |
| Whole penne^†^ | 7.43±1.98 | 29.4±7.9 | 50.6±14.8 | 34.7±30.0 | 85.6±12.4 |
| Rice^†^ | 7.55±2.86 | 3.37±2.57 | 2.73±1.24 | 3.33±5.78 | 27.0±9.73 |
| Whole rice^†^ | 3.44±0.41 | 6.73±6.00 | 19.8±34.2 | 34.8±46.7 | 63.7±1.34 |
| Biscuits^†^ | 4.07±2.11 | 58.3±27.1 | 55.9±20.7 | 30.5±52.5 | 73.9±11.0 |
| Biscuits whole grain^†^ | 3.93±2.58 | 50.5±44.8 | 41.8±36.3 | 51.2±43.7 | 63.0±21.3 |
| Wholemeal breakfast cereals^†^ | 7.46±6.03 | 66.1±23.7 | 45.1±38.6 | 60.1±51.9 | 85.5±8.17 |
| Breakfast cereals^†^ | 6.93±3.14 | 49.9±36.1 | 57.2±46.6 | 59.6±51.4 | 77.9±2.56 |

^*^Children with gluten related disorders (GRD-CH); children with obesity (OB-CH); children with allergy/intolerance to cow’s milk proteins (AICM-CH); healthy children (HE-CH).

**^†^**The existence of rows labelled with the same superscript lowercase letters for Zn-BALI values in every cereal by-product for different children groups denotes the existence of statistically significant differences (*p* < 0.05).

^#^Data from healthy children has been previously published (16).

*Table S4*.

| Food group | Zn (μg/g, ppm) | Zn-BALI in  GRD-CH (%) | Zn-BALI in  OB-CH (%) | Zn-BALI in  AICM-CH (%) | Zn-BALI in  HE-CH^#^ (%) |
| --- | --- | --- | --- | --- | --- |
| Vegetables | | | | | |
| Zucchini^†^ | 6.55±3.67 | 39.3±18.6^a^ | 30.7±28.9 | 53.1±32.3 | 60.9±19.5^a^ |
| Capsicum^†^ | 6.30±3.77 | 29.9±18.9^a^ | 32.3±29.3^b^ | 23.3±31.1^c^ | 70.7±11.5^abc^ |
| Carrot^†^ | 6.52±4.98 | 41.9±22.2^a^ | 33.8±34.3^b^ | 43.5±36.2 | 67.5±17.7^ab^ |
| Potatoe^†^ | 8.84±4.26 | 3.00±3.96^ab^ | 7.82±11.4^cd^ | 29.3±23.1^ac^ | 39.0±18.7^bd^ |
| Sweet potatoe^†^ | 11.5±6.76 | 33.4±21.2^a^ | 27.4±24.4^b^ | 33.0±26.2^c^ | 62.6±12.6^abc^ |
| Eggplant^†^ | 11.3±5.27 | 37.7±30.9^a^ | 28.0±32.1^b^ | 41.4±29.1^c^ | 72.0±10.3^abc^ |
| Onion^†^ | 8.95±6.78 | 46.6±24.4^a^ | 29.7±33.7^b^ | 29.6±31.5^c^ | 80.4±8.05^abc^ |
| Cauliflower^†^ | 8.84±5.99 | 72.8±13.6^a^ | 43.7±38.2^b^ | 40.7±35.9^ac^ | 73.0±19.0^bc^ |
| Spinach^†^ | 9-00±3.69 | 46.7±18.2^a^ | 31.0±30.0^b^ | 33.9±25.5^c^ | 66.8±9.91^abc^ |
| Garlic^†^ | 9.15±4.29 | 21.5±21.5^a^ | 21.7±23.0^b^ | 33.0±25.1 | 50.4±14.2^ab^ |
| Tomatoe^†^ | 8.09±6.13 | 18.0±16.2^a^ | 20.7±19.8^b^ | 33.3±26.2 | 52.0±18.5^ab^ |
| Cabbage^†^ | 7.86±5.77 | 39.3±25.6^ab^ | 34.6±28.3^c^ | 9.36±19.5^ad^ | 64.0±19.3^bcd^ |
| Fruits | | | | | |
| Apple^†^ | 10.8±4.65 | 57.1±30.2 | 46.4±38.5 | 42.6±28.3^a^ | 80.3±7.76^a^ |
| Banana^†^ | 7.57±5.29 | 24.9±23.2^a^ | 25.5±32.8^b^ | 34.1±32.2^a^ | 58.3±25.9^b^ |
| Orange^†^ | 7.88±4.67 | 25.9±21.4^a^ | 16.4±27.9^b^ | 35.8±27.0^c^ | 64.5±15.9^abc^ |
| Grapes^†^ | 9.95±9.40 | 36.3±26.0 | 26.0±29.4 | 30.0±26.3 | 50.6±24.8 |
| Plum^†^ | 4.28±1.82 | 35.2±26.6^a^ | 27.0±28.1 | 5.60±10.1^ab^ | 41.5±19.3^b^ |
| Peach^†^ | 4.79±2.26 | 31.2±29.4^a^ | 17.6±25.1 | 13.9±25.7^b^ | 60.8±8.11^ab^ |

^*^Children with gluten related disorders (GRD-CH); children with obesity (OB-CH); children with allergy/intolerance to cow’s milk proteins (AICM-CH); healthy children (HE-CH).

**^†^**The existence of rows labelled with the same superscript lowercase letters for Zn-BALI values in every food category for different children groups denotes the existence of statistically significant differences (*p* < 0.05).

^#^Data from healthy children has been previously published (12).
